# Riluzole Suppresses Growth and Enhances Response to Endocrine Therapy in ER+ Breast Cancer

**DOI:** 10.1210/jendso/bvad117

**Published:** 2023-09-15

**Authors:** Ayodeji O Olukoya, Hillary Stires, Shaymaa Bahnassy, Sonali Persaud, Yanira Guerra, Suman Ranjit, Shihong Ma, M Idalia Cruz, Carlos Benitez, Aaron M Rozeboom, Hannah Ceuleers, Deborah L Berry, Britta M Jacobsen, Ganesh V Raj, Rebecca B Riggins

**Affiliations:** Department of Oncology, Lombardi Comprehensive Cancer Center, Georgetown University, Washington, DC 20057, USA; Department of Oncology, Lombardi Comprehensive Cancer Center, Georgetown University, Washington, DC 20057, USA; Department of Oncology, Lombardi Comprehensive Cancer Center, Georgetown University, Washington, DC 20057, USA; Department of Oncology, Lombardi Comprehensive Cancer Center, Georgetown University, Washington, DC 20057, USA; Department of Oncology, Lombardi Comprehensive Cancer Center, Georgetown University, Washington, DC 20057, USA; Department of Biochemistry, Georgetown University, Washington, DC 20057, USA; Departments of Urology and Pharmacology, University of Texas Southwestern Medical Center at Dallas, Dallas, TX 75390, USA; Department of Oncology, Lombardi Comprehensive Cancer Center, Georgetown University, Washington, DC 20057, USA; Department of Oncology, Lombardi Comprehensive Cancer Center, Georgetown University, Washington, DC 20057, USA; Department of Oncology, Lombardi Comprehensive Cancer Center, Georgetown University, Washington, DC 20057, USA; Department of Oncology, Lombardi Comprehensive Cancer Center, Georgetown University, Washington, DC 20057, USA; Department of Oncology, Lombardi Comprehensive Cancer Center, Georgetown University, Washington, DC 20057, USA; Department of Pathology, University of Colorado Anschutz Medical Campus, Denver, CO 80045, USA; Departments of Urology and Pharmacology, University of Texas Southwestern Medical Center at Dallas, Dallas, TX 75390, USA; Department of Oncology, Lombardi Comprehensive Cancer Center, Georgetown University, Washington, DC 20057, USA

**Keywords:** estrogen receptor, riluzole, invasive lobular breast cancer, fulvestrant

## Abstract

**Background:**

Resistance to endocrine therapy in estrogen receptor–positive (ER+) breast cancer remains a significant clinical problem. Riluzole is FDA-approved for the treatment of amyotrophic lateral sclerosis. A benzothiazole-based glutamate release inhibitor with several context-dependent mechanism(s) of action, riluzole has shown antitumor activity in multiple malignancies, including melanoma, glioblastoma, and breast cancer. We previously reported that the acquisition of tamoxifen resistance in a cellular model of invasive lobular breast cancer is accompanied by the upregulation of GRM mRNA expression and growth inhibition by riluzole.

**Methods:**

We tested the ability of riluzole to reduce cell growth, alone and in combination with endocrine therapy, in a diverse set of ER+ invasive ductal and lobular breast cancer–derived cell lines, primary breast tumor explant cultures, and the estrogen-independent, *ESR1*-mutated invasive lobular breast cancer patient-derived xenograft model HCI-013EI.

**Results:**

Single-agent riluzole suppressed the growth of ER+ invasive ductal and lobular breast cancer cell lines in vitro, inducing a histologic subtype-associated cell cycle arrest (G0-G1 for ductal, G2-M for lobular). Riluzole induced apoptosis and ferroptosis and reduced phosphorylation of multiple prosurvival signaling molecules, including Akt/mTOR, CREB, and Fak/Src family kinases. Riluzole, in combination with either fulvestrant or 4-hydroxytamoxifen, additively suppressed ER+ breast cancer cell growth in vitro. Single-agent riluzole significantly inhibited HCI-013EI patient-derived xenograft growth in vivo, and the combination of riluzole plus fulvestrant significantly reduced proliferation in ex vivo primary breast tumor explant cultures.

**Conclusion:**

Riluzole may offer therapeutic benefits in diverse ER+ breast cancers, including lobular breast cancer.

Estrogen receptor–positive (ER+) breast cancer is the most commonly diagnosed cancer among women in the United States [1]. Endocrine therapies, ranging from selective estrogen receptor modulators and downregulators (SERMs, SERDs) to aromatase inhibitors, are the backbone of our current standard of care for the clinical management of ER+ breast cancers [2]. Although these treatments have significantly improved disease-free and overall survival for individuals with ER+ breast cancer, endocrine resistance remains a persistent, multifactorial problem [3]. Current efforts aim to address this problem through treatment with endocrine agents combined with other molecularly targeted therapies.

To further complicate these efforts, ER+ breast cancer is not a single disease. Invasive lobular breast cancer (ILC) is a distinct histologic subtype of breast cancer that is overwhelmingly ER+ yet has distinct genomic, transcriptomic, and proteomic features [4-6]. These distinctions have important implications for endocrine therapy response. Also, when compared to the more common invasive ductal breast cancer (IDC, invasive mammary carcinoma of no special type), ILC carries a greater risk for late recurrence (evident > 6 years after initial diagnosis) [7, 8] and responds less to the SERM tamoxifen [9, 10] and potentially the steroidal aromatase inhibitor exemestane [11]. Additionally, models of ILC are less responsive to the second-generation SERD AZD9496 than fulvestrant, while these drugs are equipotent in preclinical models of IDC [12].

Our group [13, 14] and others [12, 15-20] have identified a number of potential mechanisms that contribute to endocrine therapy resistance in ILC. We recently identified the upregulation of multiple metabotropic glutamate receptors (mGluRs, GRMs) in tamoxifen-resistant ILC cells [14]. This, coupled with other studies that directly or indirectly implicate altered amino acid metabolism and signaling in ILC preclinical models [21] and clinical disease [22, 23], led us to consider whether glutamate signaling is functionally relevant to endocrine resistance in endocrine-resistant ILC. Initially reported in melanoma [24, 25] and now other malignancies (eg, [26]), protumorigenic signaling through GRMs can be inhibited by riluzole, an oral benzothiazole-based glutamate release inhibitor that is FDA-approved for the treatment of amyotrophic lateral sclerosis (ALS). The proposed mechanism of action for riluzole within the central nervous system in ALS and in melanoma is that blocking glutamate release into the extracellular space starves GRMs of their glutamate ligand, thus functionally inhibiting them. This inhibition of the GRMs ultimately reduces glutamate excitotoxicity and inhibits tumor cell growth. In triple-negative breast cancer (TNBC), riluzole's action may not depend on GRMs [27, 28], although riluzole exerts antitumor effects [29-31]. Despite the potential of repurposing riluzole in ER+ breast cancer, especially ILC, this approach has not been a major focus to date. Therefore, this study aims to more broadly test the efficacy of riluzole, alone and in combination with multiple endocrine therapies, in a diverse set of ER+ in vitro and in vivo models enriched for ILC.

## Methods

### Cell Culture and Reagents

Several cell lines were cultured, maintained, and used in this study. These cell line models include: ER+ ILC cell lines (SUM44, LCCTam [RRID:CVCL_3424], MDA-MB-134VI [MM134] and MDA-MB-134VI long-term estrogen-deprived [LTED; MM134 LTED (RRID:CVCL_0617)], and BCK4 [RRID:CVCL_A9A5]); ER+ IDC cell lines (MCF7 and LCC9 [RRID:CVCL_DP52]) and the ER-negative (ER−) nontransformed mammary epithelial cell line MCF10A (RRID:CVCL_0598) as a control. MCF7 and LCC9; SUM44 and LCCTam; MM134 and MM134 LTED all represent pairs of parental and resistant cell lines, respectively. SUM44 and LCCTam cells were maintained under serum-free conditions in improved minimal essential media (IMEM, #A1048901, ThermoFisher, Grand Island, NY) supplemented with insulin, hydrocortisone, and other supplements as previously described [13], with the addition of 500nM 4-hydroxytamoxifen (#H7904, Sigma Aldrich, St. Louis, MO) to LCCTam cells. For selected experiments, SUM44 and LCCTam cells were maintained under serum-free conditions in a base media of Ham's F12 (#11765062, ThermoFisher) supplemented as above. MM134 cells and MM134 LTED) were maintained in IMEM supplemented with 10% fetal bovine serum (FBS) and phenol red-free IMEM (#A1048801, ThermoFisher) supplemented with 10% charcoal-cleared serum (CCS), respectively. BCK4 cells were maintained in IMEM supplemented with 10% FBS, insulin, and nonessential amino acids, as previously described [32]. MCF7 and LCC9 cells were maintained in IMEM supplemented with 5% FBS and phenol red-free IMEM supplemented with 5% CCS, respectively. MM134 and MCF7 cells were short-term hormone-deprived for selected experiments by culturing in phenol red-free IMEM supplemented with 5% CCS for 72 hours. The immortalized mammary epithelial cell line MCF10A was maintained as previously described [33]. All cell lines were authenticated by short tandem repeat (STR) profiling and regularly tested to ensure they remained free of *Mycoplasma spp*. contamination. Unless otherwise noted, general cell culture supplements and reagents were purchased from either ThermoFisher or Sigma Aldrich. Fulvestrant and riluzole were purchased from Sigma Aldrich, Tocris Bio-Techne (Minneapolis, MN), or Selleckchem (Houston, TX). Ferrostatin-1 was purchased from Selleckchem.

### Cell Proliferation Assays

On Day 0, cells were seeded in 96-well plates at the following densities: 1000 cells/well (MCF7); 2000 cells/well (LCC9, MCF10A); 10 000 cells/well (SUM44, LCCTam, MM134, MM134 LTED); and 15 000 cells/well (BCK4). Forty-eight hours later, on Day 2, cells were treated with the indicated concentration of compound(s) or dimethylsulfoxide solvent control (DMSO) for an additional 7 or 8 days, with media/compound(s) replaced on Day 5 or 6. Plates were then stained with crystal violet, dried, rehydrated, and read as previously described in [14]. Data are presented as mean ± standard error of the mean (SEM, riluzole growth curves), or median with upper/lower quartiles (effect of 10μM riluzole on cell line pairs) of % growth (vehicle = 100%) for 5 or 6 technical replicates and are representative of 2 to 4 independent biological assays. For assays of riluzole in combination with fulvestrant or tamoxifen for all cell lines except BCK4, data are processed as the mean % growth for 5 or 6 technical replicates and represent 2 to 4 independent biological assays. A representative single technical replicate's mean % growth data was then used to create a combination matrix used in SynergyFinder (RRID:SCR_019318), and the results were presented as two-dimensional (2D) surface plots. SynergyFinder uses predictive models such as highest single agent (HSA), Bliss, and zero interaction potency (ZIP) to quantify the degree of combination synergy or antagonism and outputs a synergy score. When interpreting SynergyFinder scoring, a synergy score less than −10 shows antagonistic drug interaction, a score between −10 and 10 shows an additive drug interaction, and a score greater than 10 shows a synergistic drug interaction. For the assay of riluzole in combination with fulvestrant in BCK4 cells, data are presented as median with upper/lower quartiles of % growth for 5 or 6 technical replicates and represent 2 to 4 independent biological assays.

### Cell Cycle Assays

On Day 0, cells were seeded in 6-well plates at the following densities: 150 000 cells/well (MCF7, LCC9); and 300 000 cells/well (SUM44, LCCTam, MM134, MM134 LTED, BCK4, MCF10A). Forty-eight hours later, on Day 2, cells were treated with 10μM riluzole or DMSO control for the additional indicated times before collection, fixation, staining, and cell cycle analysis by flow cytometry as described in [34]. Data are presented as mean ± SD for 3 or 4 independent biological assays.

### Annexin V Apoptosis Assays

On Day 0, cells were seeded in a 6-well plate at 300 000 cells/well (SUM44, LCCTam). Forty-eight hours later, on Day 2, cells were treated with either 10μM riluzole or DMSO solvent as a control for another 48 hours. On Day 4, cells were collected and stained with 4 µL of propidium iodide (PI) and 4 µL of annexin V conjugated with fluorescein isothiocyanate (FITC) in 100 µL of 1X binding buffer. Control cells were either left unstained or stained with either PI or annexin V dye conjugated with FITC, as described in [35]. Live (PI^−^, annexin V^−^), early apoptotic (PI^−^, annexin V^+^), late apoptotic(PI^+^, annexin V^+^), and necrotic cells (PI^+^, annexin V^−^) were quantified by flow cytometry. The PI/annexin V-FITC apoptosis detection kit was purchased from BioLegend (#640914, San Diego, CA, USA). Data are presented as mean ± SD for 3 (SUM44) or 4 (LCCTam) independent biological assays.

### Human Phospho-Kinase Proteome Profiler^TM^ Array

On Day 0, cells were seeded in a 6-well plate at 500 000 cells/well (SUM44, LCCTam). For MM134 and MM134 LTED, 1.5 million cells were seeded in 10 cm culture dishes. Forty-eight hours later, on Day 2, cells were treated with either 10μM riluzole or DMSO solvent as a control for 48 hours. On Day 4, cells were collected in 100 μL lysis buffer/well before determining total protein concentration by bicinchoninic acid (BCA) assay (#23225, ThermoFisher). According to the manufacturer's instructions, 500 micrograms of whole cell lysate were then assayed using the Human Phospho-Kinase Proteome Profiler^TM^ Array (#ARY003B [SUM44/LCCTam] and ARY003C [MM134/MM134 LTED], Bio-Techne). Array membranes were visualized using chemiluminescence detected by HyBlot CL autoradiography film (#E3018, Thomas Scientific, Swedesboro, NJ), then films were scanned and analyzed using FIJI [36]. A ratio of background-corrected intensity values for targets (phospho-kinase spots) to references (control spots) was created for each condition (DMSO and riluzole) within each cell line. Data are presented as the mean of the riluzole to DMSO ratio for 2 technical replicates from a single experiment for each cell line.

### Western Blot

SUM44 and LCCTam cells were seeded in 6-well plastic tissue culture plates at 250 000 cells/well (FAK and YES blots) or 300 000 to 600 000 cells/well (4-hydroxynonenal [4-HNE] and malondialdehyde [MDA] blots) 48 hours before treatment. The cells were treated for the time periods indicated in the figure legend. For the FAK and YES blots, the cells were treated with the control (DMSO) or drug (10µM riluzole). In the case of the MDA and 4-HNE blots, the cells were treated with control (DMSO), riluzole (10µM), or a combination of riluzole and ferrostatin-1 (10µM). After treatment, cells were lysed in radioimmunoprecipitation assay buffer (RIPA: 150mM NaCl, 50mM Tris pH 7.5, 1% Igepal CA-630, and 0.5% sodium deoxycholate) supplemented with Pierce protease and phosphatase inhibitor mini-tablets (Thermo Scientific). Protein lysates, extracted following centrifugation of the lysed cells, were mixed in a 3:1 with sample buffer (NuPAGE LDS Sample Buffer (4X) + 2-Mercaptoethanol in 2:1) and loaded onto a precast Gel (NuPAGE 4%-12% Bis-Tris Gel, Invitrogen). Proteins were transferred to nitrocellulose membranes, blocked in 5% nonfat dry milk in Tris Buffered Saline and Tween-20 [TBST; 10 mm Tris HCl, 150 mm NaCl, and 0.05% Tween-20 (pH 8.0)] at room temperature for 1 hour, then probed overnight with the following primary antibodies (diluted in TBST): phospho-FAK (1:1000, Cell Signaling Technology Cat# 3283, RRID:AB_2173659); total FAK (1:1000, Cell Signaling Technology Cat# 3285, RRID:AB_2269034); phospho-YES (1:1000, Abcam Cat# ab188319, RRID:AB_2938855); total-YES (1:500 to 1:1000, Abcam Cat# ab109265, RRID:AB_10859514); 4-HNE (1:700, Abcam Cat # ab46545, RRID:AB_722490); MDA clone −1F83 (1:200, Thermo Fisher Scientific Cat# MA5-27559, RRID:AB_2735264); SLC3A2 (1:1000, Santa Cruz Biotechnology Cat# sc-376815, RRID:AB_2938854); SLC7A11 (1:500, Abcam Cat# ab175186, RRID:AB_2722749); SLC1A5 (1:1000, Cell Signaling Technology Cat# 8057, RRID:AB_10891440); GLUD1 (1:1000, Cell Signaling Technology Cat# 12793, RRID:AB_2750880); and GPX4 (1:1000, Abcam Cat# ab125066, RRID:AB_10973901). Nitrocellulose membranes were then incubated with horseradish peroxidase (HRP)-conjugated secondary antibodies: anti-rabbit (1:2000, Cell Signaling Technology Cat# 7074, RRID:AB_2099233) or anti-mouse (1:2000, Cell Signaling Technology Cat# 7074, RRID:AB_2099233) at room temperature for 1 hour, followed by incubation in enhanced chemiluminescence from Advansta (San Jose, CA) and imaged in the Amersham imager 600 (GE Healthcare). Membranes were re-probed for beta-actin (1:2000, Cell Signaling Technology Cat# 3700, RRID:AB_2242334) or GAPDH (1:10 000 Proteintech Cat# 60004-1-Ig, RRID:AB_2107436) as a loading control.

### Cell Viability Assays

On Day 0, 300 000 to 400 000 cells of SUM44 and LCCTam were seeded in 6-well plates. Twenty-four hours later, on Day 1, cells were treated with control (DMSO), or riluzole (10μM), or a combination of riluzole and ferrostatin-1 (10μM). On Day 2, the cells were collected after a 24-hour treatment period. The collected cells were stained with trypan blue and counted using the Countess II automated cell counter (ThermoFisher). Data are analyzed using Prism 9 and presented as mean ± SD of the ratio of the cell number of the treatment groups relative to the control for 3 to 4 independent biological assays.

### HCI-013 and HCI-013EI Patient-Derived Xenograft Experiments

All animal studies were ethically conducted in accordance with our approved Institutional Animal Care and Use Committee (IACUC) protocols #2018-0005 and #2018-0006. For the comparison of time to tumor formation between HCI-013 and HCI-013EI in the presence vs absence of supplemental estrogen pellets, 5- to 6-week-old intact (nonovariectomized) female nonobese diabetic, severe combined immunodeficient mice (NOD.CB17-Prkdcscid/NCrCrl, purchased from Charles River, Wilmington, MA) were orthotopically implanted into the right fourth mammary gland with a single 1- to 3-mm^3^ patient-derived xenograft (PDX) fragment per mouse as follows: HCI-013EI (n = 6); HCI-013EI + E2 (n = 6); HCI-013 (n = 6); and HCI-013 + E2 (n = 6). The co-implantation of a 1-mg estrogen pellet under the skin on the back between the shoulder blades is designated by *+E2*. Mice were followed until measurable tumor development (by calipers), and data are presented as a survival plot with n = 6 mice per group.

For the treatment study of HCI-013EI tumors, 5- to 6-week-old intact (nonovariectomized) female mice were orthotopically implanted into the right fourth mammary gland with a single 1- to 3-mm^3^ HCI-013EI PDX fragment per mouse without estrogen supplementation, then followed until tumors reached ∼100 mm^3^ before enrollment into 1 of the 4 treatment arms: Control (n = 5); 25 mg/kg fulvestrant in castor oil subcutaneously (once per week, n = 5); 10 mg/kg riluzole orally in corn oil (5 days per week, n = 5); or the combination (n = 5) for 8 weeks. Mice were monitored for tumor growth (measured by calipers) and body weight twice per week. Tumor volumes were calculated by the modified ellipsoid formula V = 1/2(XY^2^), where X is the longest axis, and Y is the longest perpendicular axis. Tumor volume data are presented as mean ± SEM for the number of mice per treatment group. The baseline measurement represents the measurement at the point of enrollment to a treatment group based on the *a priori* tumor volume as calculated above. The subsequent measurements are those taken while the mice are on treatment at the twice per week frequency. At the study's conclusion, mice were humanely euthanized by approved American Veterinary Medical Association (AVMA) guidelines. Tumors from the treatment study were resected, weighed, formalin-fixed, and paraffin-embedded.

### Standard Immunohistochemistry Staining

Sections from formalin-fixed, paraffin-embedded tissues were deparaffinized with xylenes and rehydrated through a graded alcohol series. Heat-induced epitope retrieval (HIER) was performed by immersing the tissue sections at 98 °C for 20 minutes in LowFlex (Dako #K8005). Staining was performed following the epitope retrieval process using VectaStain Kit from Vector Labs for cleaved Caspase-3 and HRP-labeled polymer from Dako (K4001) for proliferating cell nuclear antigen (PCNA). Slides were treated with 3% hydrogen peroxide and 10% normal goat serum for 10 minutes each and exposed to primary antibodies: Caspase-3 (1:120, Cell Signaling Technology Cat# 9661, RRID:AB_2341188) and PCNA (1:1000, Santa Cruz Biotechnology Cat# sc-56, RRID:AB_628110) for 1 hour at room temperature. Slides were then exposed to appropriate biotin-conjugated secondary antibodies, Vectastain ABC reagent, and DAB chromagen (Dako) for cleaved Caspase-3 and HRP-labeled polymer and DAB chromagen (Dako) for PCNA. Slides were counterstained with hematoxylin (Fisher, Harris Modified Hematoxylin), blued in 1% ammonium hydroxide, dehydrated, and mounted with Acrymount.

### Immunohistochemistry Imaging and Analysis

Slides were scanned at 40× magnification using the Aperio GT 450, an automated digital pathology slide scanner. The whole slide scans were viewed and analyzed with QuPath-0.3.0, open-source software used for bioimage analysis [37]. The images from the Caspase-3 and PCNA slides were separated into respective project groups, and a representative image from each group was analyzed. The corresponding analysis setting was then applied to the group to ensure uniformity across all the images. First, the default stain vector was selected to deconvolute the hematoxylin and DAB stains. Next, a region of interest (ROI) for analysis was selected; in this study, the entire tumor area was established as the region of interest for this analysis. Finally, the region of interest was analyzed for positive stain detection, and the results (number positive per mm^2^) were exported as a CSV file for statistical analysis using GraphPad Prism 9. Data for both PCNA and cleaved Caspase-3 are presented as the individual and median positive cells per mm^2^ for each treatment group.

### Multiplex Immunohistochemistry Staining

HCI-013+E2 (n = 5) and HCI-013EI (n = 4) tumors from mice in our PDX maintenance colony—independent from experimental animals described above—were resected, formalin-fixed, paraffin-embedded, then sectioned for staining on the Vectra3 multispectral imaging platform (Akoya Biosciences, Marlborough, MA) using OPAL chemistry. The slides were baked at 60 °C, deparaffinized in xylene, rehydrated, washed in tap water, and incubated with 10% neutral buffered formalin for 20 minutes to increase tissue-slide retention. Epitope retrieval/microwave treatment (MWT) for all antibodies was performed by boiling slides in Antigen Retrieval buffer 6 (AR6 pH6; Akoya, AR6001KT). Protein blocking was performed using antibody diluent/blocking buffer (Akoya, ARD1001EA) for 10 minutes at room temperature. Primary antibody/OPAL dye pairings and incubation conditions for ER, progesterone receptor (PR), HER2, Ki67, and pan-cytokeratin staining are detailed in Table 1. Microwave treatment was performed to remove the primary and secondary antibodies between rounds of multiplex immunohistochemistry (IHC). Multiplex IHC was finished with microwave treatment, counterstained with spectral 4′,6-diamidino-2-phenylindole (DAPI) (Akoya FP1490) for 5 minutes, and mounted with ProLong Diamond Antifade (ThermoFisher, P36961). The order of antibody staining and the antibody/OPAL pairing was predetermined using general guidelines and the particular biology of the panel. General guidelines include spectrally separating co-localizing markers and separating spectrally adjacent dyes. Multiplex IHC was optimized by first performing singleplex IHC with the chosen antibody/OPAL dye pair to optimize signal intensity values and proper cellular expression, followed by optimizing the entire multiplex assay.

**Table 1.** Primary antibody/OPAL dye pairings and incubation conditions

	Antibody 1	Antibody 2	Antibody 3	Antibody 4	Antibody 5
**Antigen**	PR	Ki67	HER2	ER alpha	panCK
**Company**	Agilent	Agilent	Agilent	Santa Cruz	Agilent
**RRID#**	AB_2532076	AB_2142367	AB_2335701	AB_627i fi558	AB_2132885
**Species**	Mouse	Mouse	Rabbit	Mouse	Mouse
**Dilution**	1/50	1/50	1/200	1/50	1/300
**Incubation Time**	1 hour	overnight	1 hour	overnight	1 hour
**Incubation Temp.**	RT	4 °C	RT	4 °C	RT
**Control Tissue**	Breast Cancer	Tonsil	Breast Cancer	Breast Cancer	Breast Cancer
**OPAL Fluor.**	650	520	620	570	690
**OPAL Conc.**	1/140	1/30	1/160	1/125	1/30
**Antigen Retrieval**	AR6	AR6	AR6	AR6	AR6

Abbreviations: AR6, antigen retrieval buffer 6; PR, progesterone receptor; RT, room temperature.

### Multiplex Immunohistochemistry Imaging and Analysis

Slides were scanned at 10× magnification using the Vectra 3.0 Automated Quantitative Pathology Imaging System (PerkinElmer/Akoya). Whole slide scans were viewed with Phenochart (Perkin Elmer/Akoya), which allows for selecting high-powered images at 20× (resolution of 0.5m per pixel) for multispectral image capture. Multispectral images of each xenograft tissue specimen were captured in their entirety. Multispectral images were unmixed using spectral libraries built from images of single stained tissues for each reagent using the inForm Advanced Image Analysis software (inForm 2.4.6; PerkinElmer/Akoya). A selection of 10 to 15 representative multispectral images spanning all 9 tissue sections was used to train the inForm software (tissue segmentation, cell segmentation, and phenotyping tools). All the settings applied to the training images were saved within an algorithm to allow the batch analysis of all the multispectral images particular to each panel. Data are presented as the overall mean ± SD of % marker positivity for all tumors.

### Fluorescence Lifetime Imaging Instrumentation

A modified Olympus FVMPERS (Waltham, MA) microscope equipped with a Spectra-Physics Insight X3 (Milpitas, CA) laser and FastFLIM (ISS, Champaign, IL) acquisition card were used to image the cancer samples by fluorescence lifetime imaging (FLIM). The samples were excited by two-photon excitation at 740 nm using a 20× air objective (LUCPLFLN 0.45NA, Olympus), and the emitted fluorescence was collected using the DIVER (Deep Imaging Via Enhanced Recovery) detector assembly equipped with a FastFLIM card for lifetime imaging. The pixel dwell time was fixed at 20 µs, and the field of view was 318.8 µm (Zoom = 2X) at 256X256 pixels. To increase the signal-to-noise ratio, 16 frames were integrated. The data from each pixel were recorded and analyzed using the SimFCS software (available from the Laboratory for Fluorescence Dynamics, University of California, Irvine, CA). The raster scanning was done using the Olympus software, and the images were collected using the FLIMBox/FastFLIM system in passive mode [38].

The samples (5 µm thick) were imaged using the homebuilt DIVER (Deep Imaging via Enhanced Recovery) microscope [39], a homebuilt modified detector based on an upright configuration. The details of this microscope have been described elsewhere [40, 41]. Briefly, this microscope uses a forward detection scheme and a large area photon counting detector (R7600P-300, Hamamatsu), having a higher photon collection efficiency due to the large cone angle of detection. A combination of filters capable of separating the blue wavelength (400-500 nm) fluorescence was used for FLIM imaging of NADH [42]. The phasor plot is calibrated using Rhodamine 110 in water which has a mono-exponential lifetime of 4.0 ns.

### FLIM Phasor Analysis [38, 43, 44]

The fluorescence intensity decays collected at each pixel of the image were transformed to the Fourier space, and the phasor coordinates were calculated using the following relations:


(1) $$g_{i,\;j}(\omega )=\int_{0}^{\infty }\;I(t)\cdot Cos(n\omega t)dt/\int_{0}^{\infty }\;I(t)dt\;$$

(2) $$s_{i,\;j}(\omega )=\int_{0}^{\infty }\;I(t)\cdot Sin(n\omega t)dt/\int_{0}^{\infty }\;I(t)dt\;$$

where *g_i, j_(ω)* and *s_i, j_(ω)* are the X and Y coordinates of the phasor plot, respectively, and *n* and *ω* are the harmonic numbers and the angular frequency of excitation, respectively. The transformed data were then plotted in the phasor plot so that the data from each pixel is transformed to a point in the phasor plot [45-47].

The fractional intensity distribution between free and protein-bound NADH was calculated based on a two-component analysis of the phasor plot [38, 48] and then converted to concentration ratio based on the quantum yield of the 2 species [49]. The higher free/bound NADH ratio is representative of increased glycolysis [43].

### Primary Breast Tumor Explant Cultures

Patient-derived explants (PDEs) from 5 ER+ primary breast tumors were processed and cultured as described in [50]. PDEs were treated with 100nM fulvestrant, 10μM riluzole, the combination, or solvent control (DMSO) for 48 hours before formalin fixation, paraffin embedding, sectioning, and staining for PCNA (1:1000, Santa Cruz Biotechnology Cat# sc-56, RRID:AB_628110), cleaved caspase 3 (1:300, Cell Signaling Technology Cat# 9661, RRID:AB_2341188), and Ki67 (1:500, Abcam Cat# ab16667, RRID:AB_302459). Stained sections were then visualized and scored as described in [50]. Data are presented as change relative to vehicle (set to 0) for each PDE.

### Statistical Analysis

Statistical analyses were performed using GraphPad Prism 9.0 (San Diego, CA) at α ≤ 0.05, except for riluzole/fulvestrant and riluzole/4-hydroxytamoxifen combination experiments, which were analyzed by SynergyFinder [51]. Single-agent riluzole experiments were analyzed by nonlinear regression ([inhibitor] vs normalized response), and response to 10μM riluzole in endocrine therapy sensitive/resistant cell line pairs (SUM44 vs LCCTam and MCF7 vs LCC9) was compared by Mann-Whitney test. Riluzole/fulvestrant and riluzole/4-hydroxytamoxifen combination experiments were analyzed using the Bliss, zero interaction potency (ZIP), and highest single agent (HSA) methods [52] in SynergyFinder. Cell cycle and Annexin V apoptosis assays were analyzed by two-way analysis of variance (ANOVA) followed by Sidak's multiple comparisons tests. Cell viability assays for riluzole and ferrostatin-1 were analyzed by two-way ANOVA followed by Tukey's multiple comparisons test. Staining for each marker in primary breast tumor explant cultures was analyzed by one-sample *t* test vs 0 (vehicle). In the xenograft experiment comparing time to tumor formation between HCI-013 and HCI-013EI in the presence vs absence of supplemental estrogen pellets, data were analyzed by log-rank (Mantel-Cox) test. In the xenograft experiment testing fulvestrant, riluzole, the combination, or control in HCI-013EI, tumor volume, and mouse body weight data were analyzed by mixed-effects analysis followed by Dunnett's multiple comparisons tests at each time point vs control. Tumor weight at the endpoint and PCNA and cleaved caspase-3 were analyzed by Browne-Forsyth and Welch ANOVA, followed by Dunnett's T3 multiple comparison tests. Partial response, stable disease, and progressive disease were calculated using RECIST 1.1 criteria [53]. Multiplex IHC data were analyzed by the Mann-Whitney test.

## Results

Riluzole has shown antitumor activity in preclinical models of multiple cancers, including melanoma, glioblastoma, and breast cancer [25-31]. In several (but not all) of these reports, riluzole-mediated growth inhibition is attributed to increased expression of metabotropic glutamate receptors (mGluRs, GRMs). We previously reported that acquisition of tamoxifen resistance in a cellular model of invasive lobular breast cancer (ILC [13],) is accompanied by the upregulation of GRM mRNA expression and growth inhibition by riluzole [14]. Here, our goal was to test the efficacy of riluzole more broadly, alone and in combination with multiple endocrine therapies, in a diverse set of ER+ in vitro and in vivo models enriched for ILC.

### Riluzole Suppresses Growth in ER+ Breast Cancer Cell Lines

**Figure 1.**
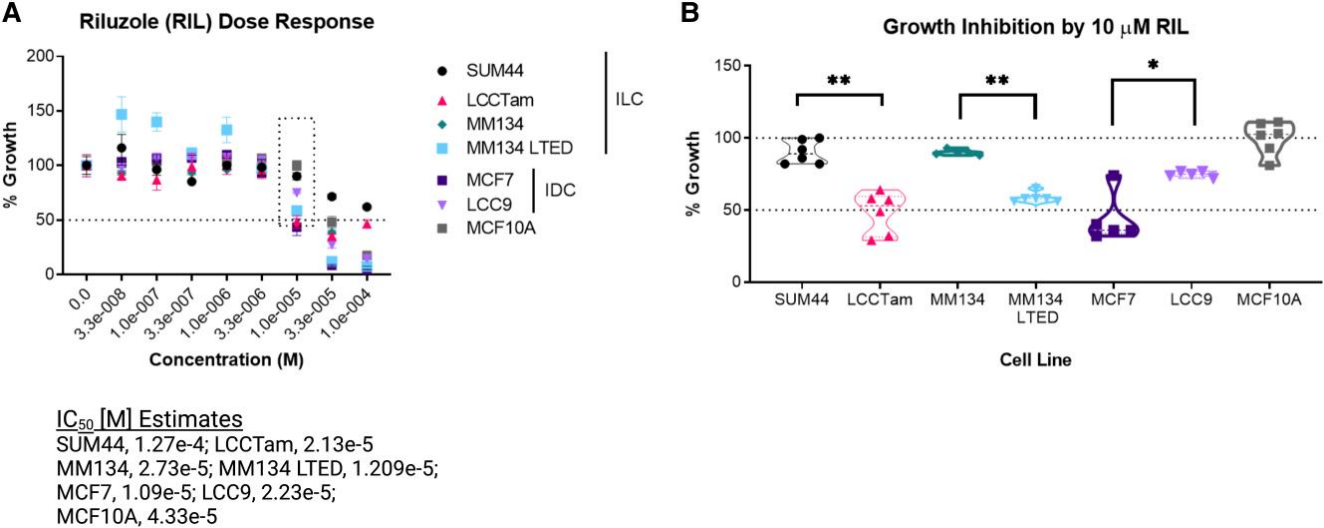
Growth suppression of ER+ breast cancer cell lines by riluzole. A, Cells seeded in 96-well plates were treated with the indicated concentrations of riluzole (RIL, 33nM to 100μM) or DMSO control for 7 to 8 days prior to staining with crystal violet. Data are presented as mean % growth ± standard error of the mean (SEM) of % growth (vehicle = 100%) for 5 or 6 technical replicates and represent 2 to 4 independent biological assays. The dotted line box indicates data re-graphed in panel B. Data were analyzed by nonlinear regression ([inhibitor] vs normalized response), yielding the following IC_50_ [M] estimates: SUM44, 1.27e-4; LCCTam, 2.13e-5; MM134, 2.73e-5; MM134 LTED, 1.209e-5; MCF7, 1.09e-5; LCC9, 2.23e-5; MCF10A, 4.33e-5. B, Relative response to 10μM RIL re-graphed from panel A (dotted line box). Data are presented as median % growth with upper/lower quartiles of % growth (vehicle = 100%) for 5 to 6 technical replicates and represent 2 to 4 independent biological assays. For the SUM44/LCCTam, MM134/MM134 LTED, and MCF7/LCC9 cell line pairs, data were compared by the Mann-Whitney test. **P = .002, **P = .0043, and *P = .024 respectively. Dashed lines denote 50% (panels A and B) and 100% growth (panel B).

We performed dose-response assays of riluzole (33 to 100μM) in 4 ILC- and 2 IDC-derived cell lines and the nontransformed ER− breast epithelial cell line MCF10A, using crystal violet staining as a proxy for total cell number [14] (Fig. 1A). Nonlinear regression analysis calculated the riluzole IC_50_ for all 6 cell lines to be ∼10 to 100μM, consistent with published studies in other malignancies. Direct comparison of growth inhibition by 10μM riluzole in 3 endocrine-responsive and -resistant cell line pairs (Fig. 1B) confirmed [14] that the tamoxifen-resistant ILC cell line LCCTam, and the MM134 LTED cells, were significantly more responsive to riluzole than their parental counterparts SUM44 and MM134 (Mann-Whitney test, **P = .002 and *P* = .0043 respectively). This was not the case for the MCF7/LCC9 IDC cell line pair [54], in which MCF7 cells showed greater riluzole-mediated growth inhibition (*P = .024) than fulvestrant-resistant/tamoxifen-cross-resistant LCC9 cells. However, MCF10A nontransformed cells were not growth-inhibited by 10μM riluzole vs DMSO control.

The presence vs absence of steroid hormones and estrogenic compounds in growth media (eg, phenol red, serum) can influence the response of ER+ cell lines to growth inhibition by small molecules. The SUM44/LCCTam cell line pair is cultured in serum-free media, but a phenol red-containing base (IMEM, 10 mg/L), while MCF7 and MM134 cells are cultured in phenol red-containing, IMEM supplemented with 5% FBS. LCC9 cells and MM134 LTED are maintained in hormone-replete conditions. As such, experiments presented in Fig. 1 performed under hormone-replete conditions were repeated under hormone-deprived conditions (Supplementary Fig. S1 [55]). While individual differences within cell lines were observed, hormone deprivation—reduced phenol red media for SUM44/LCCTam (Ham's F12, 1.2 mg/L) or phenol red-free IMEM supplemented with 5% CCS for MCF7 and MM134—did not consistently enhance or impair riluzole-mediated growth inhibition.

### Riluzole Induces a Histologic Subtype-Associated Cell Cycle Arrest

**Figure 2.**
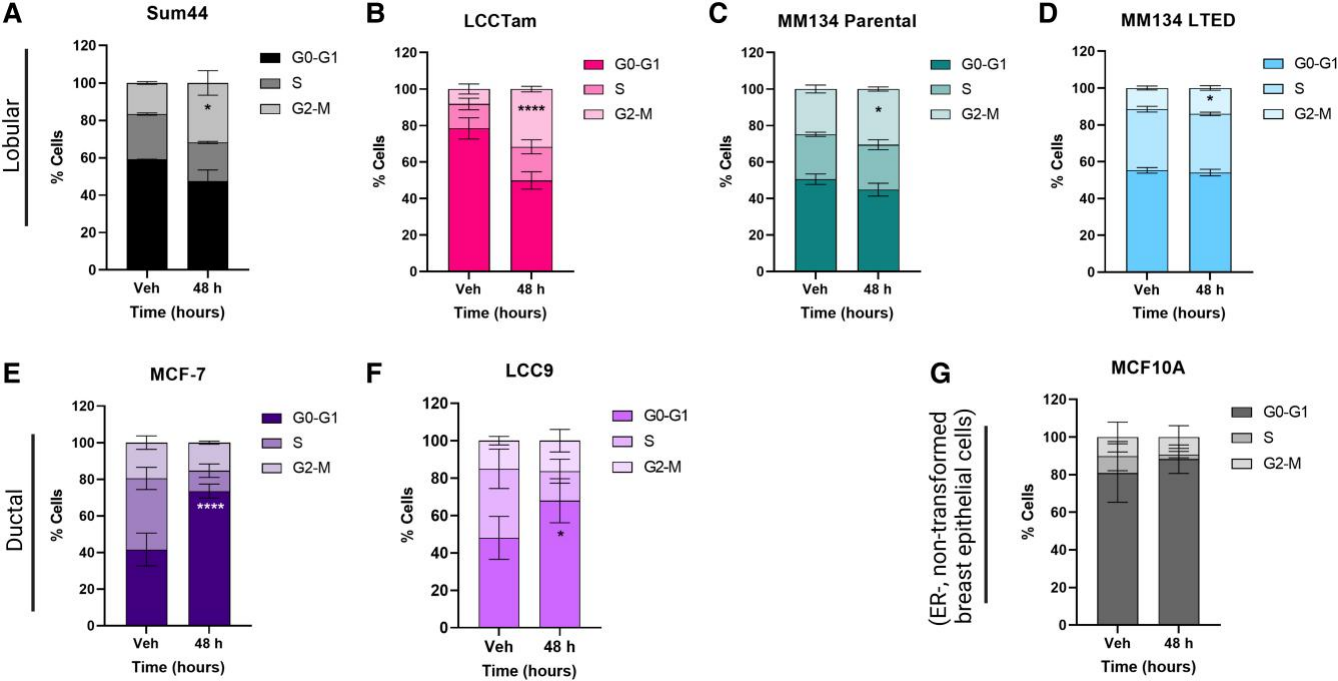
Riluzole induces a histologic subtype-associated cell cycle arrest (A-D, ILC; E-F, IDC; G, nontransformed cell control). Cells seeded in 6-well plates were treated with 10μM riluzole or DMSO control (vehicle, Veh) for the indicated times prior to collection, fixation, staining, and cell cycle analysis. Data are presented as mean % cells ± SD for 3 or 4 independent biological assays and analyzed by two-way ANOVA followed by either Sidak's (single time point) or Dunnett's (multiple time points) multiple comparisons tests. SUM44: *P = .018. LCCTam: ****P < .0001. MM134: *P = .011. MM134 LTED: *P = .05. MCF7: ****P < .0001. LCC9: *P = .015. MCF10A: not significant.

To corroborate the cell proliferation assay results, we tested riluzole's effect on cell cycle progression (Fig. 2). All ILC cell lines (including BCK4, a third model of ER+ ILC [32], Supplementary Fig. S2 [55]) showed a significant accumulation of cells in the G2-M phase (two-way ANOVA followed by Sidak's multiple comparisons test, see figure legends). However, both IDC-derived cell lines showed a significant accumulation of cells in the G0-G1 phase, while nontransformed MCF10A cells showed no significant cell cycle arrest in response to riluzole. Together with the results presented in Fig. 1 and Supplementary Fig. S1 [55], these data suggest that while all ER+ breast cell lines tested are growth-inhibited by riluzole, ILC cells preferentially undergo G2-M arrest while IDC cells arrest in G0-G1.

### Riluzole Inhibits Phosphorylation of Prosurvival Signaling Molecules and Fak/Src Kinases

**Figure 3.**
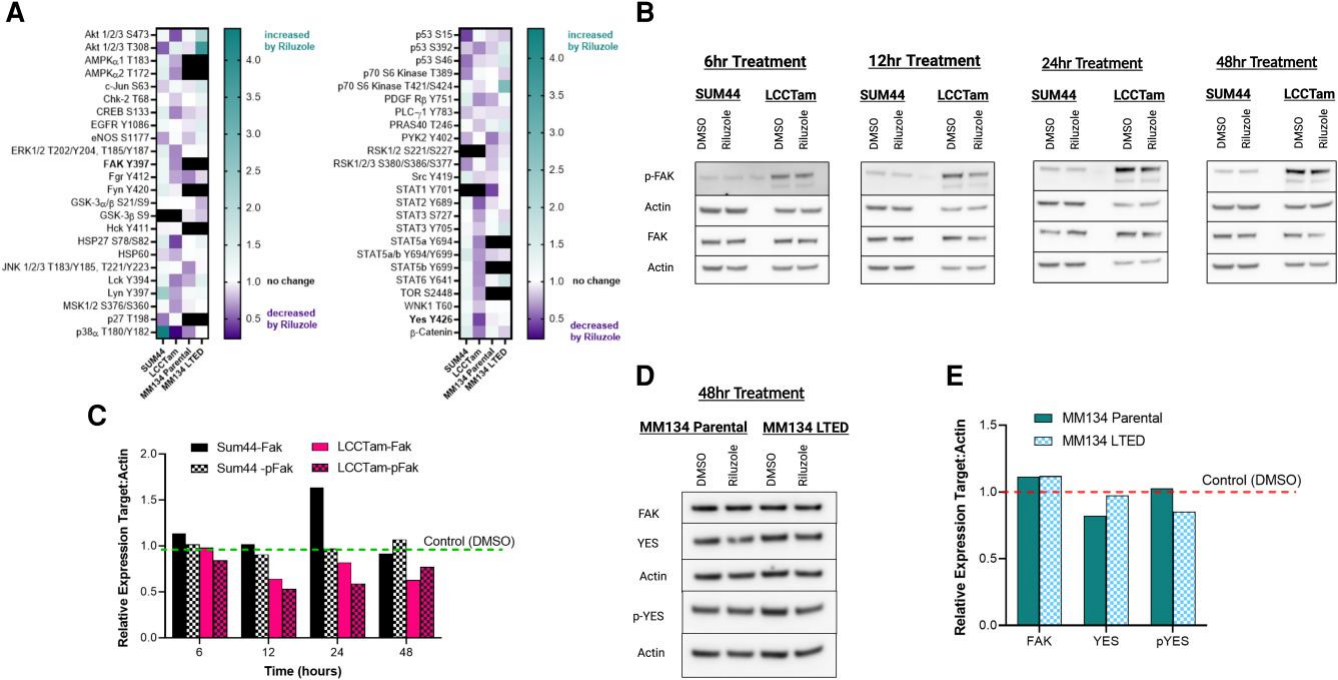
Riluzole inhibits phosphorylation of prosurvival signaling molecules and Fak/Src kinases. A, Cells seeded in 6-well plates were treated with 10μM riluzole or DMSO control (vehicle, Veh) for 2 days prior to collection, lysis, and processing, then assayed using the Human Phospho-Kinase Proteome Profiler^TM^ Array. A ratio of background-corrected intensity values for targets (phospho-kinase spots) to references (control spots) was created for each condition (DMSO and riluzole) within each cell line. Black squares indicate absence of the indicated phospho-antibody from the array used for that cell line pair. Data are presented as the geometric mean of the riluzole: DMSO ratio for 2 technical replicates from a single experiment. B, SUM44, and LCCTam cells seeded in 6-well plates were treated with 10μM riluzole or DMSO control for several time points (6,12, 24, and 48 hours). Cells were collected, lysed, and Western blot analysis was performed to test for expression and phosphorylation of Fak Y397. The data are presented as images showing expression levels. C, Quantification analysis of Fak and *P*-Fak protein band density from Western blot in Fig. 3B. D, MM134, and MM134 LTED, cells seeded in 6-well plates were treated with 10μM riluzole or DMSO control for 48 hours. Cells were collected, lysed, and Western blot analysis was performed to test for expression of Fak, as well as expression and phosphorylation of Yes Y426. E, Quantification analysis of Fak, Yes, and *P*-Yes protein band density from Western blot in Fig. 3D.

To identify molecular signaling events accompanying riluzole-mediated growth inhibition in the 2 ILC endocrine therapy sensitive/resistant cell line pairs, we used the Human Phospho-Kinase Proteome Profiler Array to detect changes in 43 phosphorylation sites across 40 different kinases or substrates (Fig. 3A). In SUM44 cells, riluzole reduced phosphorylation of mutant p53 [56] (S15, S46, and S392), Akt T308, p27 (T198), p70 S6 kinase (T389), and RSK1/2/3 (S380/386/377). In LCCTam cells, riluzole reduced phosphorylation of markedly more sites in kinases and substrates. Notable inhibition of Akt/mTOR (Akt S437, TOR S2448, PRAS40 T246), CREB (Msk S376/360, CREB S133), Fak/Src family kinase (Lyn Y397, Yes Y426, Fak Y397), and STAT (multiple) signaling pathways was observed in LCCTam vs SUM44 cells. In the MM134/MM134 LTED cell line pair, suppression of kinases and substrates was more limited, although inhibition of Fak/Src family kinases Fgr (Y412, both cell lines), Lck (Y394, MM134), Pyk2 (Y402, MM134), as well as Stat1 (Y701, MM134) and Stat5a/b (Y694/699, both cell lines) was observed. Prior studies in melanoma and glioblastoma have shown that riluzole inhibits Akt phosphorylation and that riluzole combined with mTOR inhibition can synergistically decrease xenograft growth [26, 57]. However, to our knowledge, inhibition of Fak/Src family kinases by riluzole has not been previously reported. Therefore, to further validate the inhibition of selected Fak/Src kinases observed from the Human Phospho-Kinase Proteome Profiler Array, we performed a Western blot analysis on both cell line pairs treated with riluzole for expression and phosphorylation of Fak (Y397) and Yes (Y426). The results show markedly higher baseline Fak Y397 phosphorylation and confirmed reduced expression and phosphorylation of Fak Y397 in the LCCTam cells vs no change to a slight increase in Fak phosphorylation in SUM44 cells (Fig. 3B and 3C). However, we could not validate a change in Yes expression or phosphorylation at Y426 in SUM44 or LCCTam cells (Supplementary Fig. S3A [55]). By contrast, Yes Y426 phosphorylation was modestly higher in MM134 LTED cells, while expression and phosphorylation of Yes (but not Fak) were modestly inhibited in MM134 and MM134 LTED, respectively (Fig. 3D and 3E).

### Riluzole Can Induce Apoptosis and Ferroptosis and Alters Expression of Glutamate Transporters and Metabolic Enzymes

**Figure 4.**
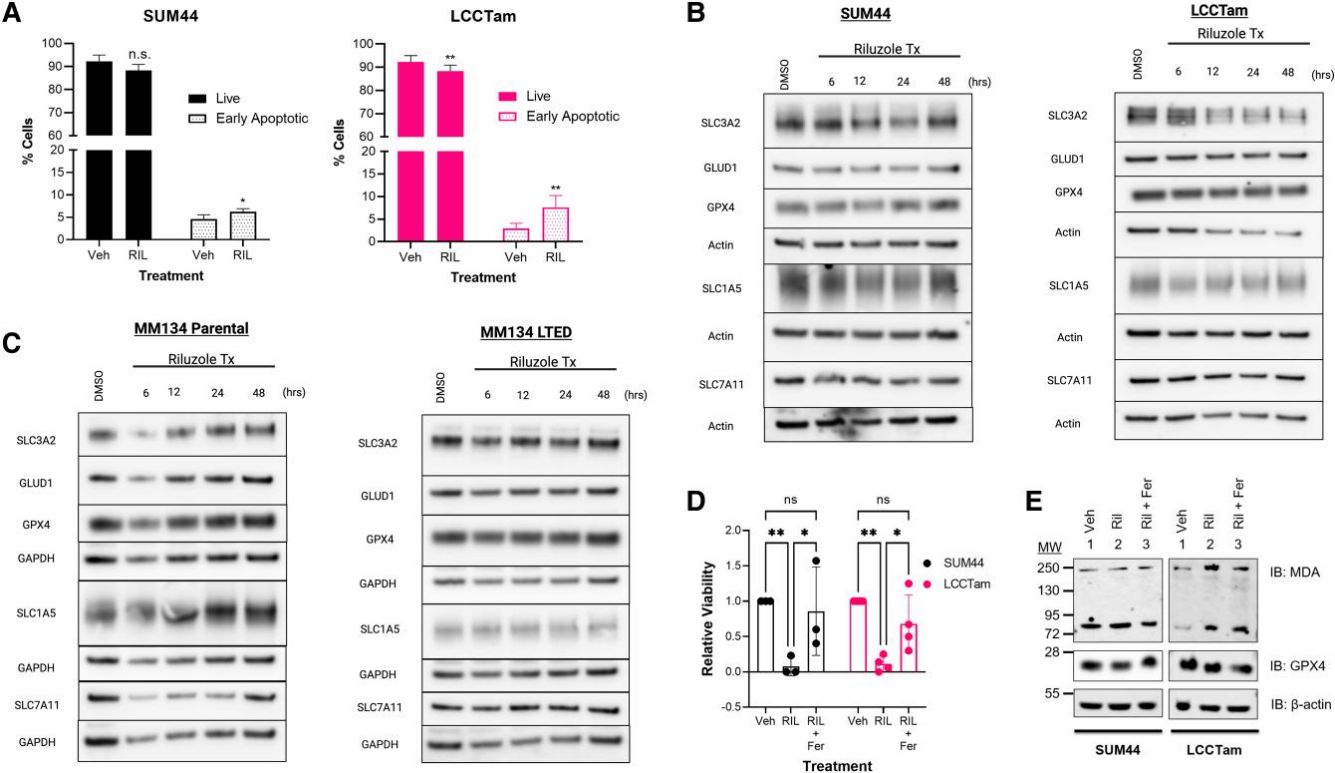
Riluzole can induce apoptosis and ferroptosis, and it alters expression of glutamate transporters and metabolic enzymes. A, Cells seeded in 6-well plates were treated with 10μM riluzole or DMSO control (vehicle, Veh) for 2 days prior to staining with Annexin V and PI. The percent of live cells (PI^−^, annexin V^−^) and early apoptotic (PI^−^, annexin V^+^) cells are shown. Data are presented as mean % cells ± SD for 3 (SUM44) or 4 (LCCTam) independent biological assays and analyzed by two-way ANOVA followed by Sidak's multiple comparisons test (*P = .021, **P = .04 (live) and **P = .011). B and C, SUM44 and LCCTam (B), and MM134 and MM134 LTED (C), cells seeded in 6-well plates were treated with 10μM riluzole or DMSO control for several time points (6,12, 24, and 48 hours). Cells were collected, lysed, and Western blot analysis was performed to test for expression of SLC1A5, SLC3A2, SLC7A11, GLUD1, and GPX4. The data are presented as images showing expression levels. D, SUM44 and LCCTam cells were seeded in 6-well plates. Twenty-four hours later, cells were treated with control (DMSO), riluzole (10μM), or a combination of riluzole and ferrostatin-1 (10 uM). 24 hours after treatment, the cells were collected, stained with trypan blue, and counted. Data are presented as mean ± SD of the ratio of the cell number of the treatment groups relative to the control for 3 or 4 independent biological assays and analyzed by two-way ANOVA followed by Tukey's multiple comparison test (SUM44, [**P = .005, *P = .016]; LCCTam, [**P = .002, *P = .043]). E, SUM44, and LCCTam cells seeded in 6-well plates were treated with control (DMSO), riluzole (10µM), or a combination of riluzole and ferrostatin-1 (10µM) for 48 hour. After treatment, cells were collected, lysed, and Western blot analysis was performed to test for expression of the malondialdehyde (MDA) byproduct of lipid peroxidation, and GPX4. The data are presented as images showing expression levels.

Given the wider array of prosurvival kinases inhibited by riluzole in the SUM44/LCCTam cell line pair, we performed Annexin V assays to measure apoptosis. LCCTam cells showed a significant increase in the percent of cells in early apoptosis when treated with riluzole (Fig. 4A and Supplementary Fig. S3C [55], two-way ANOVA followed by Sidak's multiple comparisons tests, **P < .001). However, there was a less robust increase in early apoptotic SUM44 cells (two-way ANOVA followed by Sidak's multiple comparisons test, *P < .0211). These data are consistent with those presented in Fig. 1B, where LCCTam cells were significantly more growth-inhibited by riluzole than SUM44 cells.

In addition to apoptosis, PI3K-Akt-mTOR pathway inhibition has been shown to induce iron-dependent cell death, or ferroptosis [58]. Fak signaling downstream of the glutamate/cystine antiporter (SLC3A2/SLC7A11 heterodimer, or X_c_^−^), which can be inhibited by riluzole, has also been implicated in ferroptosis [59]. We performed Western blot analysis for a panel of solute carriers (SLCs) and 2 glutamate metabolic enzymes (glutamate dehydrogenase 1 [GLUD1], and glutathione peroxidase 4 [GPX4]) with links to ferroptosis in the SUM44/LCCTam and MM134/MM134 LTED cell line pairs (Fig. 4B and 4C, and Supplementary Fig. S3B [55]). In SUM44 cells, 12 to 24 hours of riluzole treatment modestly inhibited expression of SLC3A2 and SLC7A11, as well as SLC1A5, a neutral amino acid transporter best known for importing glutamine that also has pH-dependent glutamate/glutamine antiporter activity [60]. In LCCTam cells, SLC and loading control expression decreased from 12 to 48 hours while GLUD1 and GPX4 remained constant, resulting in a net two-fold increase of these enzymes. In MM134 cells riluzole led to a transient decrease in SLC3A2 and SLC7A11 expression at 6 hours, but there were no consistent alterations in MM134 LTED cells.

Riluzole-mediated downregulation of SLC3A2, SLC7A11, and/or SLC1A5, coupled with stabilization of GLUD1 and/or GPX4 as a compensatory mechanism (discussed in [61]) could suggest the induction of ferroptosis, so we performed a viability assay after treating Sum44 and LCCTam cells with vehicle (DMSO), or riluzole or a combination of riluzole and ferrostatin-1 (inhibitor of ferroptosis). The results showed that riluzole reduces cell viability in both SUM44 and LCCTam, and the addition of ferrostatin-1 reverses the observed reduction (Fig. 4D). To further substantiate this observation, we performed a Western blot analysis on the SUM44/LCCTam cell line pair treated with vehicle (DMSO) or riluzole, or a combination of riluzole and ferrostatin for 48 hours, then probed for malondialdehyde (MDA) and 4-hydroxynonenal (4-HNE), which are both byproducts of ferroptosis. MDA levels were increased in LCCTam cells treated for 48 hours with riluzole. Conversely, ferrostatin-1 decreased the riluzole-induced MDA (Fig. 4E). Riluzole slightly increased the levels of 4-HNE in LCCTam cells, whereas the combined treatment of riluzole and ferrostatin-1 reduced the levels of riluzole-induced 4-HNE (Supplementary Fig. S3D [55]).

### Riluzole, in Combination With Endocrine Therapies, Leads to Additive Suppression of ER+ Breast Cancer Cell Line Growth in Vitro

**Figure 5.**
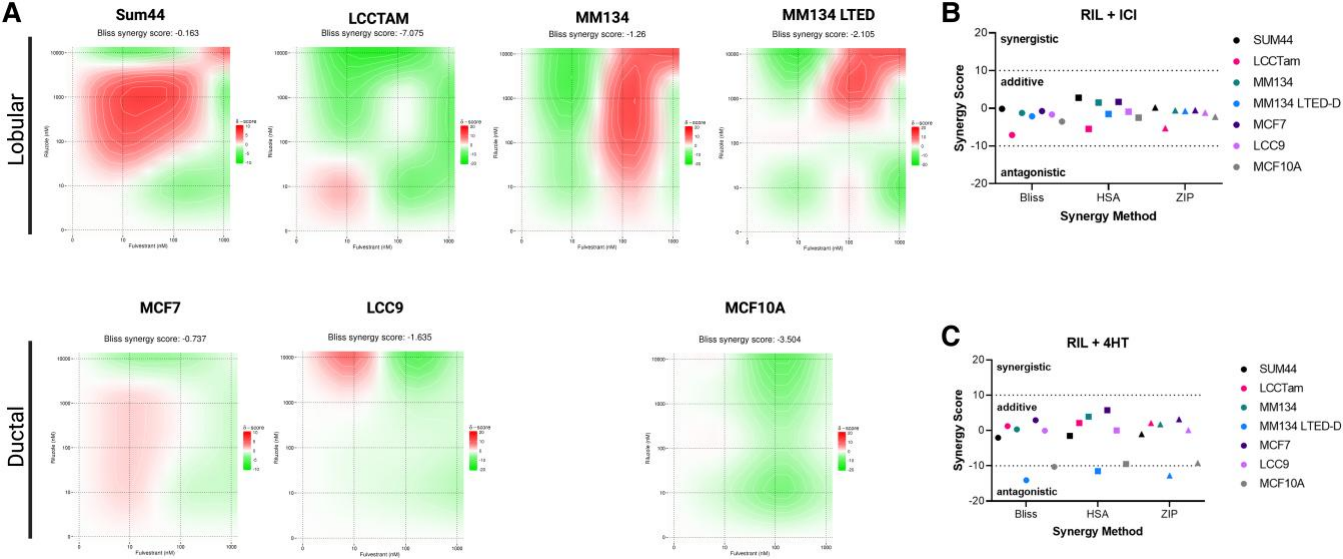
Additive suppression of ER+ breast cancer cell line growth by riluzole in combination with endocrine therapies. A, Cells seeded in 96-well plates were treated with 1μM fulvestrant, 10μM riluzole (RIL), the combination, or DMSO control (vehicle, Veh) for 7 to 8 days prior to staining with crystal violet. Data are processed as the mean % growth for 5 or 6 technical replicates and represent 2 to 4 independent biological assays. The mean % growth data of a representative single technical replicate was then used to create a combination matrix used in SynergyFinder, and the results were presented as 2D surface plots. The SynergyFinder scores are shown at the top of the plots, highlighting the level of synergy. B and C, Graphical representation of the synergy scores from the riluzole/fulvestrant (B) and riluzole/4-hydroxytamoxifen (4HT) (C) combination using 3 models: Bliss, highest single agent (HSA), and zero interaction potency (ZIP).

Endocrine therapies ranging from SERMs and SERDs to aromatase inhibitors represent the standard of care for the clinical management of ER+ breast cancers [2]. Therefore, we tested riluzole's activity in combination with the SERD fulvestrant or SERM tamoxifen (4-hydroxytamoxifen) in ILC- and IDC-derived ER+ breast cancer cell lines and the ER− nontransformed breast epithelial cell line MCF10A as a negative control. These experiments were conducted under hormone-replete conditions. To determine the possible relational effect of the drug combinations, we used SynergyFinder, a web-based tool for interactive analysis and visualization of multidrug and multidose response data [51]. Based on the synergy finder scoring, the combination of fulvestrant and riluzole showed additive benefits in nearly all tested cell lines (Fig. 5 and Supplementary Fig. S4A [55]). The representative synergy map of the bliss model highlights the synergistic and antagonistic dose regions in red and green, respectively, and the overall synergy score indicated at the top (Fig. 5A, > 10 = synergy, 10 to −10 = additive, < −10 = antagonistic). Examination of the other synergy models provided similar synergy scores to the bliss model in Fig. 4A, which supports the notion that the combination of fulvestrant and riluzole is additive (Fig. 5B). The synergy analysis of the combination of riluzole and tamoxifen resulted in scores that indicated an additive interaction in all the cell lines except for MM134 LTED and MCF10A (Fig. 5C). MM134 LTED cells were only inhibited by the lowest concentration of hydroxytamoxifen and not by the higher concentrations (Supplementary Fig. S4B [55]). On the other hand, riluzole significantly inhibited growth, therefore, the drugs’ opposing effects likely account for the combination's antagonistic effect. Despite these exceptions, these data suggest that the combination of endocrine therapy and riluzole, in most cases, can additively suppress the growth of a variety of ER+ breast cancer cell line models.

Single-agent riluzole inhibits tumor growth in vivo, but the combination with fulvestrant is not better than fulvestrant alone in the HCI-013EI ILC PDX model. PDXs are an important, clinically relevant alternative to 2D culture models for preclinical testing of combination therapies. The HCI-013 PDX model was established from a 53-year-old woman with metastatic, multi-therapy-resistant ER+/PR+/HER2− ILC by serial passage through intact female NOD scid gamma (NSG) mice supplemented with a 1-mg estrogen (E2, 17β-estradiol) pellet [15]. The HCI-013EI (estrogen-independent) variant was established by 2 weeks of in vitro culture of cells from HCI-013 tumors under hormone-deprived conditions, then reimplanted into intact female NSG mice without estrogen supplementation [62]. Both models harbor the clinically relevant *ESR1* activating mutation Y537S, with the HCI-013EI variant reported as having a more abundant variant allele fraction of Y537S.

**Figure 6.**
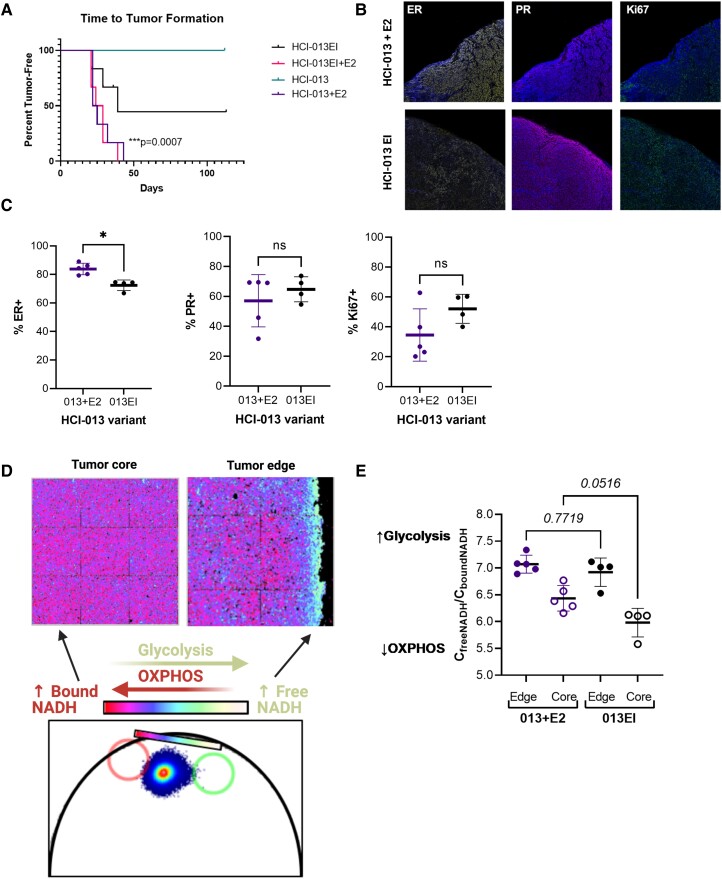
Characterization of tumor growth, hormone receptor expression, and metabolic state for HCI-013 vs HCI-013EI ILC PDX models. A, Tumor latency for HCI-013 and HCI-013EI patient-derived xenografts (PDXs) with or without estrogen (E2) supplementation. Mice (6 mice per group) were orthotopically implanted with a 1- to 3-mm^3^ PDX fragment, then followed until measurable tumor development (by calipers). Data are presented as percent tumor free and were analyzed by log-rank (Mantel-Cox) test. ***P = .0007. B, Representative images of ER, PR, and Ki67 staining from HCI-013+E2 and HCI-013EI tumors. C, Semi-quantitative analysis of multiplex IHC (mIHC) staining for ER, PR, and Ki67 from HCI-013+E2 (n = 5) and HCI-013EI (n = 4) tumors from mice independent of those for whom tumor latency is shown in panel A. Data are presented as overall mean ± SD of % marker positivity for 5 to 13 fields per tumor. D, Representative schematic of fluorescence lifetime imaging microscopy (FLIM) analysis of cellular metabolism at the tumor core and edge. Images are pseudo-colored based on the phasor plot (below) where more protein-bound and more free NADH phasor positions are indicated by red and cyan circles, respectively, and the color scheme chosen reflects more bound NADH in purple and more free NADH in cyan. E, Quantification of FLIM in HCI-013 + E2 vs HCI-013EI tumor cores and edges. Tumor edges were strongly glycolytic in both HCI-013 + E2 and HCI-013EI, but tumor cores were preferentially in an oxidative phosphorylated state in HCI-013EI.The data were analyzed by one-way ANOVA (*P* < .0001) followed by Tukey's multiple comparisons test. Each symbol indicates the mean C_free NADH_/C_bound NADH_ for 16 fields of view of the tumor edge or core in an individual tumor (n = 4-5 tumors per PDX line).

To directly compare the responsiveness to, and dependency on, supplemental estrogen of HCI-013 vs HCI-013EI, 6 intact (5-6 weeks old) severe combined immunodeficient (SCID) female mice per group were orthotopically implanted with a single 1- to 3-mm^3^ PDX fragment, then tumor growth and development was monitored (Fig. 6A). In the presence of supplemental estrogen pellets, HCI-013 and HCI-013EI exhibited a 100% tumor take rate, with a median time to tumor formation of 23.5 and 26.5 days, respectively. However, in the absence of supplemental estrogen pellets, HCI-013 PDX fragments were unable to form tumors out to 113 days postimplantation, and HCI-013EI PDX fragments exhibited a 50% tumor take rate, with a median time to tumor formation of 39 days (log-rank Mantel-Cox test, ***P = .0007). These data suggest that supplemental estrogen is necessary for HCI-013 tumor formation and beneficial but not necessary for HCI-013EI tumor formation in SCID mice.

We characterized an independent set of HCI-013+E2 (estrogen supplemented, n = 5) and HCI-013EI (not estrogen supplemented, n = 4) tumors with respect to hormone receptor (ER and PR), HER2, and proliferative marker Ki67 expression using OPAL chemistries on the Vectra3 multispectral imaging platform (Fig. 6B and 6C). This approach captured heterogeneity in marker expression between and within tumors. Overall, percent ER positivity (% ER+) was significantly lower in HCI-013EI vs HCI-013+E2 tumors (Mann-Whitney test, *P = .032), consistent with a prior report that Y537S mutant ER protein expression can be lower than wild type ER [63]. However, overall percent PR and Ki67 positivity were not significantly different between these PDX variants. No HER2 staining was detected (data not shown).

An important feature of endocrine-resistant breast cancer is dysregulated metabolism, with published studies showing increased dependency on glutamine [64] and other amino acids [65]. Advanced imaging techniques like fluorescence lifetime imaging (FLIM) take advantage of the natural autofluorescence of biomolecules, including the reduced form of nicotinamide adenine dinucleotide (NADH, a key output of cellular metabolism). FLIM coupled with phasor analysis permits resolution of bound vs free NADH, which correlates with oxidative phosphorylation vs glycolytic metabolism, respectively [38, 66]. Using FLIM, we examined the cellular metabolism of HCI-013+E2 and HCI-013EI tumors. We observed that cells were mainly glycolytic at the edge of both HCI-013+E2 and HCI-013EI tumors. However, cells at the core of HCI-013EI tumors were preferentially in an oxidative phosphorylated state as opposed to a more glycolytic state observed in cells within the core of the HCI-013+E2 tumors (Fig. 6D and 6E, one-way ANOVA [*P* < .0001] followed by Tukey's multiple comparisons test [*P* = .0516]).

**Figure 7.**
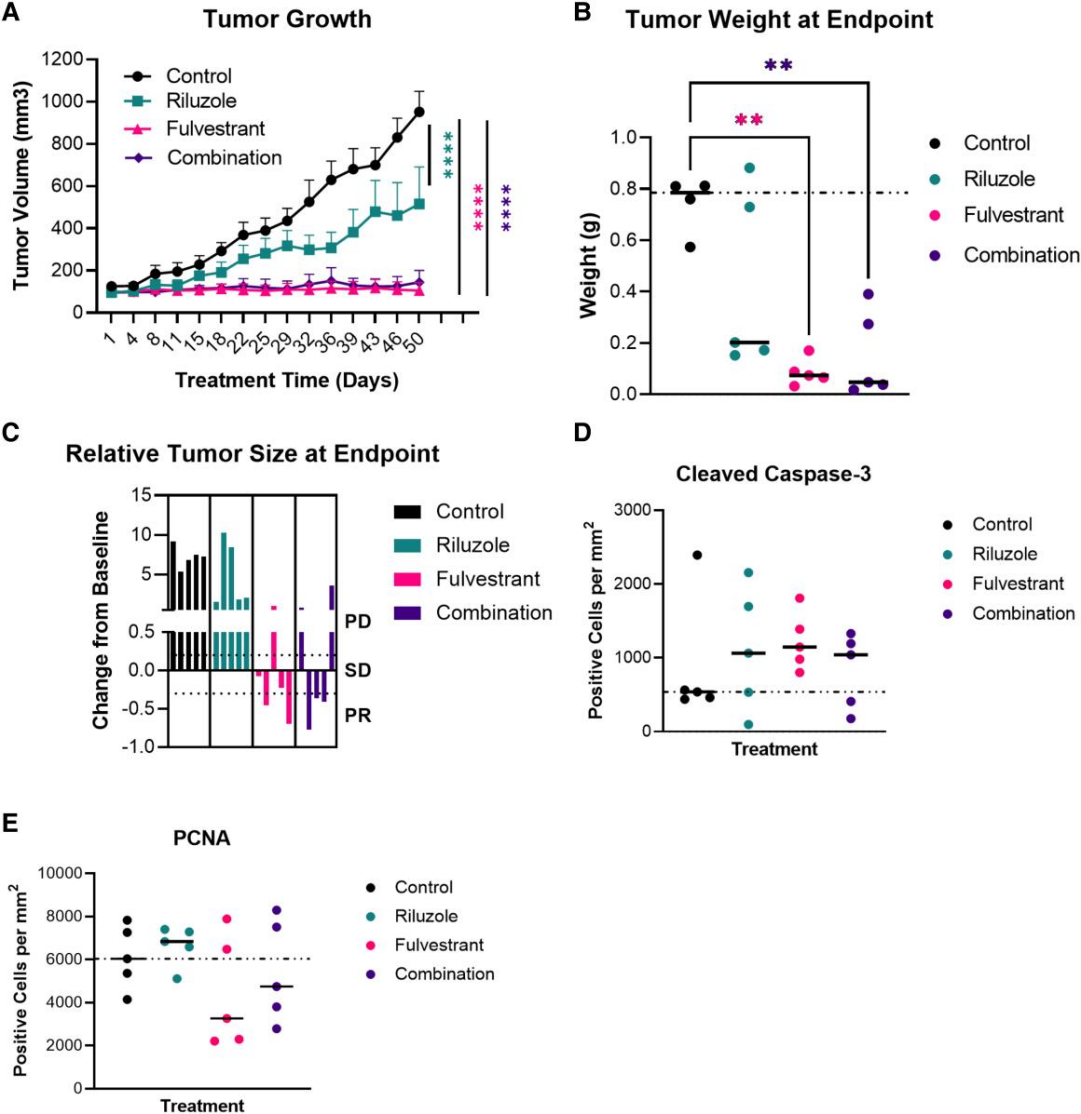
Single-agent riluzole inhibits tumor growth in vivo, but the combination with fulvestrant is not better than fulvestrant alone in the HCI-013EI ILC PDX model. A, Forty-eight mice were orthotopically implanted with a 1- to 3-mm^3^ HCI-013EI PDX fragment without E2 supplementation, then followed until tumors reached ∼100 mm^3^ before enrollment into 1 of 4 treatment arms: control (n = 5), fulvestrant (n = 5), riluzole (n = 5), or the combination (n = 5). Mice were monitored for tumor growth (measured by calipers) and body weight twice per week. Data are presented as mean tumor volume ± SEM, and were analyzed by mixed-effects analysis followed by Dunnett's multiple comparisons tests at each timepoint vs control. B, At the end of the study, tumors were collected and weighed. The graph illustrates the summary of the collected data, which were analyzed using Browne-Forsyth and Welch ANOVA followed by Dunnett's T3 multiple comparisons tests. C, Graph showing relative tumor size at endpoint according to RECIST 1.1 criteria. It shows that 2 of 5 tumors in the fulvestrant group and 3 of 5 in the combination group achieved partial response. Abbreviations in Figure 7C graph: PD, progressive disease; PR, partial response; SD, stable disease. D and E, The tumors collected from each treatment group were formalin-fixed, paraffin-embedded, sectioned, and stained with proliferating cell nuclear antigen (PCNA) and Caspase-3 by IHC. These antibodies served as a proxy for proliferation and apoptosis, respectively. The stained samples were analyzed, and the data were presented graphically in D for PCNA, and E for Caspase-3.

We selected the HCI-013EI (not estrogen supplemented) PDX variant to test the antitumor activity of fulvestrant, riluzole, or the combination relative to vehicle control. Forty-eight 5- to 6-week-old intact SCID female mice were orthotopically implanted with a single 1- to 3-mm^3^ HCI-013EI PDX fragment without E2 supplementation, then followed until tumors reached ∼100 mm^3^ before enrollment into 1 of 4 treatment arms: control (n = 5), fulvestrant (n = 5), riluzole (n = 5), or the combination (n = 5) (Fig. 7A and Supplementary Fig. S5A [55]). Relative to control, the single-agent riluzole, fulvestrant, and the combination significantly slowed the tumor volume. However, the level of tumor growth inhibition relative to control varied among the 3 groups. The riluzole group showed about 50% inhibition, whereas similar effects but greater inhibition (∼90%) were observed in the fulvestrant and combination groups. (Fig. 7A, mixed effect analysis followed by Tukey's multiple comparisons tests). Analysis of tumor weight at the endpoint for the treatment groups further supported the observed difference in tumor volume. The mean weights of the fulvestrant, riluzole, and combination groups were lower than the control group. However, only the fulvestrant and combination groups showed statistically significant differences **(**Fig. 7B, Browne-Forsyth and Welch ANOVA followed by Dunnett's T3 multiple comparisons tests) and were not different from each other. Analysis of relative tumor size at endpoint according to RECIST 1.1 criteria [53] shows that 2 of 5 tumors in the fulvestrant group and 3 of 5 in the combination group achieved partial response (Fig. 7C). We then performed immunohistochemistry to stain for proliferating cell nuclear antigen (PCNA) and Caspase-3 as a proxy for proliferation and apoptosis, respectively (Figs. 7D and 7E, and Supplementary Fig. S5C [55]). Although not significant, the mean positive cells per mm^3^ of Caspase-3 for the fulvestrant, riluzole, and combination group were each higher than the control group (Fig. 7D). For the PCNA staining, expectedly, the fulvestrant and combination group had lower mean positive cells per mm^3^. However, surprisingly, the mean positive stained cells per mm^3^ for the riluzole group was higher than the control group (Fig. 7E). Finally, analysis of mouse body weights between the treatment groups showed no significant differences. As seen in Supplementary Fig. S5B [55], the slope of the graphs for each treatment group is close to zero. Altogether, these data show that single-agent riluzole has a significant inhibitory effect on HCI-013EI tumor volume, and with fulvestrant already highly effective against this PDX model, combination treatment does not provide additional benefit.

### Riluzole Plus Fulvestrant Significantly Inhibits Proliferation in Primary Breast Tumor Explant Cultures

**Figure 8.**
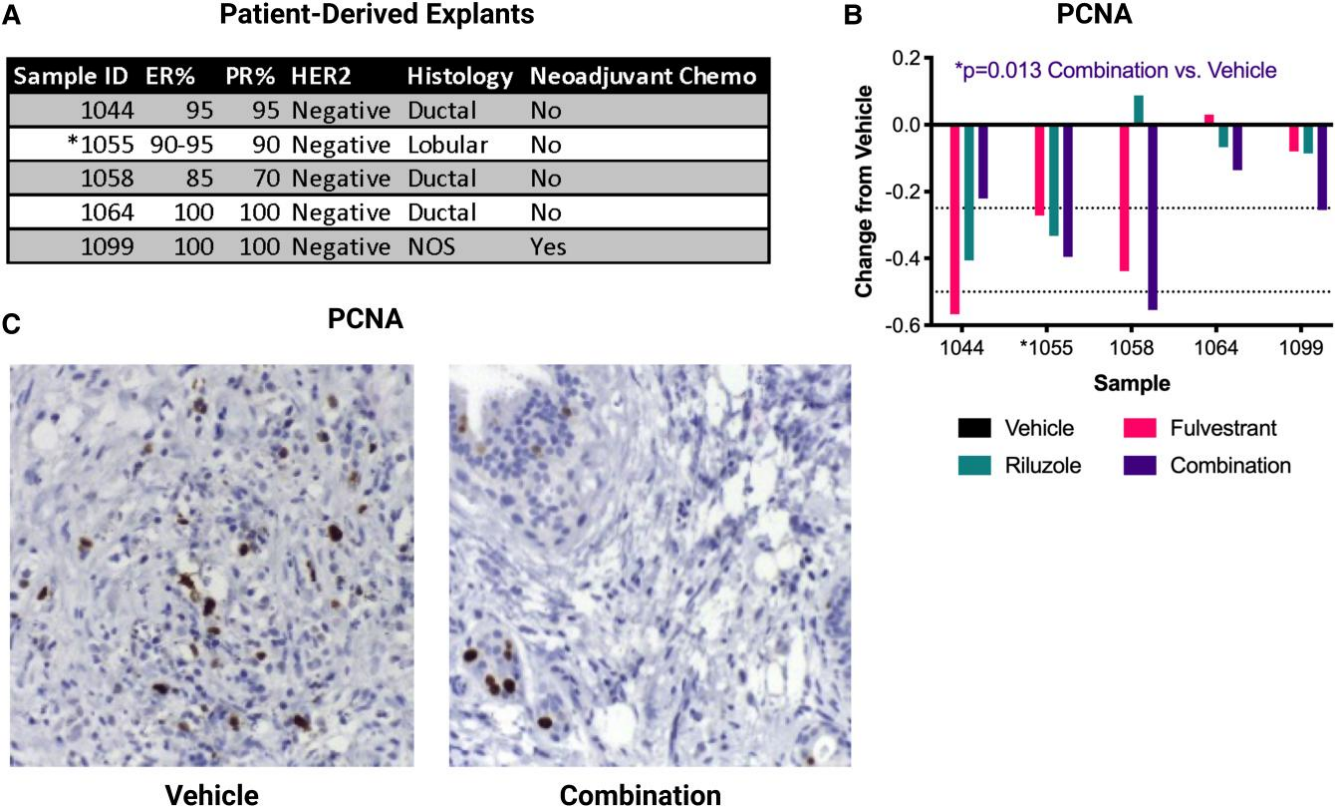
Riluzole plus fulvestrant significantly inhibits proliferation in primary breast tumor explant cultures. A, Pathologic data for 5 patient-derived explants (PDEs). ER, PR, and Ki67% are from the initial surgical specimen, and NOS = not otherwise specified. *denotes the PDE for which representative images are shown in panel C. B, PDEs were treated with 100nM fulvestrant, 10μM riluzole, the combination, or DMSO control (vehicle) for 2 days prior to formalin fixation, paraffin embedding, sectioning, and staining for PCNA by IHC. Data are presented as change relative to vehicle (set to 0) for each explant and analyzed by one-sample *t* test vs 0 (vehicle). *P = .013 Vehicle vs Combination. *denotes the PDE for which representative images are shown in panel C. C, Representative images of PCNA and Caspase-3 staining from PDE #1055 (ILC).

Patient-derived explants (PDEs) provide another preclinical strategy to test combination therapies. These short-term cultures of surgical samples maintain the local tumor microenvironment and capture interperson heterogeneity [50]. We tested the efficacy of fulvestrant, riluzole, or the combination vs solvent control (DMSO) in 5 PDEs from ER+/PR+/HER2−negative primary tumors (Fig. 8A). Using PCNA staining as a proxy for cell proliferation, the combination of riluzole plus fulvestrant significantly reduced PCNA (Figs. 8B and 8C, one-sample *t* test vs 0 [vehicle], *P = .013 vehicle vs combination), with 4 of 5 PDEs showing better growth inhibition by the combination than either drug alone and the greatest effect seen in the ILC PDE. Staining for cleaved caspase 3 suggested a modest induction of apoptosis by either drug alone or the combination in some of the PDEs (Supplementary Fig. S6 [55]), but this was not statistically significant. Together with the results presented in Figs. 5 and 6, and accompanying supplementary figures, these data suggest that combining fulvestrant and riluzole may offer improved therapeutic benefits in some ER+ breast cancers.

## Discussion

We tested the efficacy of riluzole, alone and in combination with multiple endocrine therapies, in a diverse set of ER+ in vitro and in vivo models enriched for ILC. Single-agent riluzole suppressed the growth of ER+ ILC and IDC cell lines in vitro, by inducing a histologic subtype-associated cell cycle arrest (G0-G1 for IDC, G2-M for ILC). In the tamoxifen-resistant ILC-derived LCCTam model, riluzole induced apoptosis and ferroptosis, and reduced phosphorylation of multiple prosurvival signaling molecules, including Akt/mTOR, CREB, and Fak/Src family kinases. Furthermore, in combination with either fulvestrant or 4-hydroxytamoxifen, riluzole additively suppressed ER+ breast cancer cell growth in vitro, and significantly reduced proliferation in ex vivo primary breast tumor explant cultures.

The increased combinatorial efficacy of riluzole and fulvestrant we observe across diverse cell line models of ER+ breast cancer in vitro is recapitulated ex vivo using PDEs. In the HCI-013EI PDX experiment, however, tumor volume after ∼7 weeks of treatment was not significantly different in combination- vs fulvestrant-treatment groups. Single-agent riluzole had significant activity at 10 mg/kg, a dose consistent with that used in preclinical studies of ALS and other motor neuron diseases (eg [67, 68]). We attribute this lack of additive or synergistic effect in our in vivo study partly to the very strong response of the HCI-013EI PDX to single-agent fulvestrant as seen in this experiment (∼90% inhibition) and reported by others [45, 46]. In the ex vivo PDE experiment, the combination of riluzole and fulvestrant was highly effective and more robust than either agent alone, with 80% (4/5) of primary tumor explants showing significant growth inhibition by the combination as measured by a reduction in PCNA (Fig. 7). Improved combinatorial efficacy in the PDEs may also be due to the lower concentration of fulvestrant used in these studies (100nM). Additionally, riluzole bioavailability is variable, leading to mixed efficacy in preclinical and clinical studies. For example, in preclinical studies of triple-negative breast cancer [30] and glioblastoma [26], single-agent riluzole does not have significant antitumor activity in vivo, whereas in our study and preclinical studies of melanoma [24, 25, 69] it does. However, in Fig. 6B and 6C, 2 of 5 riluzole-treated tumors showed no reduction in tumor weight or relative tumor size vs control-treated tumors, which could be due to inconsistent bioavailability. Serum levels of the drug vary widely in ALS patients receiving the drug, and in a phase II trial for advanced melanoma, circulating riluzole concentrations had marked interpatient variability [70]. The riluzole prodrug troriluzole [71] appears to offer better bioavailability by reducing first-pass metabolism by CYP1A2, leading to sustained ∼0.3μM to 1.8μM plasma concentrations of active drug in a recent phase Ib study that combined troriluzole (BHV-4157) with nivolumab in advanced solid tumors [72]. These and other riluzole analogs will be important to explore alone and in combination with endocrine therapy in ER+ breast cancer.

Riluzole induces apoptosis and ferroptosis concomitant with reduced phosphorylation of multiple prosurvival signaling molecules. That these include components of the Akt/mTOR signaling pathway (Akt S437, TOR S2448, PRAS40 T246) is not surprising since riluzole has been shown to inhibit Akt phosphorylation and synergize with mTOR inhibition in melanoma and glioblastoma models [26, 57]. However, Fak/Src kinase family members (eg, Yes and Fak) have not, to our knowledge, been previously implicated in riluzole action. Our data (Fig. 3) show that Fak and Yes expression and phosphorylation are inhibited by riluzole in the SUM44/LCCTam and MM134/MM134 LTED cell line pairs, respectively. Multiple Src/Fak kinase family members play critical roles in cell survival, invasion, and migration. Additionally, Fak has been previously implicated in endocrine therapy resistance [73, 74]. Being functionally E-cadherin-negative, ILC is highly resistant to anoikis (a form of cell death induced by extracellular matrix detachment) [75] and dependent upon a rewired actin cytoskeleton and constitutive actomyosin contractility (reviewed in [76]). Consistent with this, expression of activated Src and Fak—both of which can drive resistance to anoikis [77]—are significantly higher in ILC vs atypical lobular hyperplasia [78]. In parallel, ILC may exhibit increased sensitivity to ferroptosis inducers. Ferroptosis is cell density–dependent, and cells cultured in low-density conditions with fewer cell-cell contacts (a defining feature of ILC) are highly vulnerable to ferroptosis caused by inhibition of GPX4. On the other hand, E-cadherin-mediated intercellular interactions suppress ferroptosis via activation of neurofibromin 2 (NF2, [79]). The specific contribution of Fak/Src kinases to riluzole-mediated growth inhibition, apoptosis, and ferroptosis in ILC remains to be explored and will be a component of future studies, as will its role in the additive or synergistic growth suppression achieved by the combination of riluzole and endocrine therapy. Nevertheless, it is tempting to speculate that agents (like riluzole) that broadly target rewired cytoskeletal regulatory pathways *and* induce ferroptosis may be particularly effective against ILC.

This study has limitations that are important to consider. First, hormone-responsive ER+ models, particularly of ILC, are limited. In this study, we used 3 of the 4 well-established ER+, hormone-responsive ILC lines [75], the ILC-derived HCI-013EI PDX model [15, 62], and 1 of the 5 PDEs originated from ILC. In all ILC models we tested except for the HCI-013EI PDX, riluzole plus fulvestrant provided greater growth inhibition than single-agent fulvestrant. Expansion of the PDE approach is an important strategy for rapidly diversifying the repertoire of preclinical ILC models [80] to test this and other novel endocrine therapy combinations, essential for a breast cancer subtype that has a significantly greater risk of late recurrence, and worse response to tamoxifen and the second-generation SERD AZD9496 [12]. Second, riluzole's multiple proposed or confirmed mechanisms of action—from inhibition of signaling through GRMs [24] and glutamate release via the glutamate/cystine antiporter SLC7A11 or X_c_^−^ [81], to blockade of voltage-gated sodium channels [82], inhibition of internal ribosome entry site (IRES)-mediated protein synthesis [26], attenuation of RNA polymerase III complex assembly [83], and inhibition of Wnt/β-catenin signaling [84]—present a challenge to readily identifying patients who would benefit most from the drug. Some of this variability is cancer type–specific, with riluzole action tightly coupled to GRM expression in melanoma [24] and, to some extent, glioblastoma [26], but not in triple-negative breast cancer [27]. While reasonably well-defined in the central nervous system, the interconnected pathways of glutamate release, uptake, and signaling remain understudied in epithelial cells and their pathologies. We posit that future preclinical studies of riluzole in this context (epithelial tumors generally, and ER+ ILC more specifically) should address these limitations.

## Conclusions

Our data suggest that riluzole may offer therapeutic benefits in diverse ER+ breast cancers, including ILC, and support optimization and further investigation of riluzole and its combinations in this setting.

## Data Availability

All data generated or analyzed during this study are included in this published article and its supplementary information files (published in the Zenodo repository, https://doi.org/10.5281/zenodo.7600716).

## Abbreviations

2D: two-dimensional
4-HNE: 4-hydroxynonenal
ALS: amyotrophic lateral sclerosis
ANOVA: analysis of variance
CCS: charcoal-cleared serum
DMSO: dimethylsulfoxide
E2: estradiol
ER+: estrogen receptor–positive
FBS: fetal bovine serum
FITC: fluorescein isothiocyanate
GLUD1: glutamate dehydrogenase 1
GPX4: glutathione peroxidase 4
HRP: horseradish peroxidase
IDC: invasive ductal breast cancer
ILC: invasive lobular breast cancer
IMEM: improved minimal essential media
LTED: long-term estrogen-deprived
MDA: malondialdehyde
mGluR/GRM: metabotropic glutamate receptor
MM134: MDA-MB-134VI cell line
NSG: NOD scid gamma
PCNA: proliferating cell nuclear antigen
PDE: patient-derived explant
PDX: patient-derived xenograft
PI: propidium iodide
SCID: severe combined immunodeficiency
SEM: standard error of the mean
SERD: selective estrogen receptor downregulator
SERM: selective estrogen receptor modulator
SLC: solute carrier
